# Development and Psychometric Validation of the Brain Rot Scale: Measuring Digital Content Overconsumption Among Generation Alpha and Generation Z

**DOI:** 10.3390/ejihpe15120262

**Published:** 2025-12-18

**Authors:** Mamdouh Mahmoud Mostafa, Ashraf Ragab Ibrahim, Mohamed Ali Nemt-allah, Safaa Zaki Arafa, Amina Ahmed Hassan, Mamdouh Mosaad Helali

**Affiliations:** 1 Mental Health Department, Faculty of Education, Al-Azhar University, Cairo 11765, Egypt; mamdouhbadawy.197@azhar.edu.eg; 2 Educational Psychology and Statistics Department, Faculty of Education, Al-Azhar University, Dakahlia 35822, Egypt; ashrafibrahem.26@azhar.edu.eg; 3 College of Science, King Faisal University, Al-Ahsa 31982, Saudi Arabia; szarafa@kfu.edu.sa (S.Z.A.); aahassan@kfu.edu.sa (A.A.H.); 4 The National Research Center for Giftedness and Creativity, King Faisal University, Al-Ahsa 31982, Saudi Arabia

**Keywords:** brain rot, digital addiction, Generation Alpha, Generation Z, measuring

## Abstract

Brain rot refers to the cognitive decline and mental exhaustion resulting from excessive consumption of low-quality, short-form digital content, particularly affecting Generation Alpha and Generation Z. This study developed and validated the Brain Rot Scale (BRS) to assess digital content overconsumption among digital natives aged 8–24 years. A two-phase design employed separate Egyptian samples for exploratory factor analysis (EFA; *n* = 403) and confirmatory factor analysis (CFA; *n* = 897). The initial 21-item Arabic scale underwent principal axis factoring with promax rotation, guided by parallel analysis. Following iterative item deletion, a 14-item scale (BRS-14) emerged with three factors: Attention Dysregulation (6 items), Digital Compulsivity (5 items), and Cognitive Dependency (3 items), accounting for 35.114% of common variance. Confirmatory factor analysis demonstrated excellent fit (CFI = 0.988; TLI = 0.985; RMSEA = 0.031 [0.023, 0.039]; SRMR = 0.040), with standardized loadings ranging from 0.667 to 0.758 (*p* < 0.001). The scale showed excellent reliability (*ω* = 0.900, *α* = 0.899), with subscale reliabilities from 0.759 to 0.857. Convergent validity was established (CR > 0.70, AVE > 0.50). Factor intercorrelations (0.636–0.671) supported a hierarchical model where a general Brain Rot factor explained 62.9–69.9% of first-order variance. The BRS-14 provides a psychometrically sound instrument for assessing problematic digital consumption patterns among contemporary youth populations.

## 1. Introduction

The rapid evolution of digital media platforms has fundamentally transformed content consumption patterns, particularly among younger generations who have grown up immersed in the digital ecosystem. The emergence of short-form video platforms such as TikTok and Instagram has introduced unprecedented changes in how individuals engage with digital content, creating new behavioral phenomena that challenge traditional understanding of media consumption and its psychological implications. These platforms have introduced novel consumption patterns including binge-scrolling—the rapid, continuous consumption of sequential short-form videos—characterized by diminished self-control and temporal disorientation (Park & Jung, 2024). The integration of infinite scroll mechanisms has intensified platform dependence, with users frequently exceeding intended usage durations while experiencing reduced cognitive engagement (Y. Wang, 2023).

Contemporary digital consumption is particularly pronounced among Generation Alpha (born after 2010) and Generation Z (born 1995 to 2010), who represent a fundamental paradigm shift in digital media engagement. These cohorts are distinguished from previous generations by their status as true digital natives who integrate technology seamlessly into daily routines from early childhood (Dunas & Vartanov, 2020; Šramová & Pavelka, 2023). Unlike previous generations, who engaged in more passive, scheduled media consumption, these cohorts demonstrate intentional, selective, and highly interactive engagement patterns, with a pronounced preference for mobile-first, on-demand content across platforms such as TikTok and Instagram (Cifuentes-Ambra et al., 2023; Laor & Galily, 2022).

The scale of digital engagement among these generations has reached unprecedented levels, with profound implications for cognitive development and behavioral patterns. Screen time among Generation Alpha and Generation Z has escalated dramatically, with the average 17–19-year-old spending approximately 6 h daily on mobile digital devices (Manwell et al., 2022). These digital natives demonstrate complex multitasking behaviors, frequently using smartphones as second screens while engaging with other media content (Yeşilyurt & Karaduman, 2025). Current research indicates that Generation Z students spend 9 h or more per day on smartphones and social media, with 70% acknowledging internet addiction (Ahmed, 2019). While media consumption remained stable until 2010, it increased significantly by 2014, particularly among teenagers (Zdanowicz et al., 2020).

This excessive digital consumption has given rise to the culturally significant concept of “brain rot,” which has emerged as a metaphor for the perceived cognitive decline and mental exhaustion associated with overconsumption of low-quality digital content, particularly among younger generations (Özpençe, 2024; Yousef et al., 2025). Brain rot refers to the deterioration of mental or intellectual abilities due to overconsumption of trivial, repetitive, or low-value online content. This phenomenon reflects growing societal concerns about how constant exposure to trivial online content can erode attention spans, critical thinking, and emotional well-being in the digital age (Abdo & Pego-Fernandes, 2025; Sage, 2025). The cultural resonance of this term, exemplified by its designation as Oxford’s Word of the Year 2024, reflects widespread anxieties about technology’s impact on cognitive health and meaningful communication (Yazgan, 2025).

The concerns surrounding excessive digital consumption are increasingly supported by empirical research revealing significant neurobiological and cognitive consequences. Existing neuroscience research reveals concerning impacts that may be associated with excessive digital stimulation on developing brains, with studies demonstrating significant cognitive, emotional, and behavioral consequences. Research has shown that prolonged screen exposure has been associated with impaired learning and memory acquisition, reduced academic performance, and alterations in brain structure and function (Neophytou et al., 2019). Excessive digital media use correlates with increased rates of anxiety, depression, and behavioral problems in youth (Manwell et al., 2022), while chronic overuse may disrupt normal brain maturation processes, particularly in regions responsible for emotional regulation and executive function (Nivins et al., 2024; Small et al., 2022; Yousef et al., 2025).

The addictive potential of digital platforms is consistent with strategic neurobiological targeting mechanisms. Specifically, social media platforms employ intermittent reinforcement schedules, algorithmically delivering unpredictable rewards such as likes and notifications. This mechanism may potently activate the brain’s dopamine reward pathways (Lindström et al., 2021). These variable reinforcement patterns appear to engage midbrain limbic dopamine systems, particularly through social prediction error mechanisms in the ventral tegmental area, where dopamine neurons may encode differences between expected and received social rewards (Solié et al., 2022). The unpredictable nature of these social rewards may create heightened anticipation and reinforce repeated engagement behaviors, establishing patterns similar to those observed in substance addiction (De et al., 2025; J. Wang & Wang, 2025).

Concurrent with these neurobiological changes, digital devices are increasingly replacing traditional cognitive processes, potentially leading to measurable neuroplastic changes in brain structure and function. The phenomenon of cognitive offloading, defined as the use of external tools or actions to reduce mental effort in memory tasks (Risko & Gilbert, 2016), has become particularly relevant. While this strategy enhances immediate task performance, particularly under high cognitive load conditions (Morrison & Richmond, 2020), research demonstrates complex long-term consequences for memory consolidation. Specifically, cognitive offloading can diminish memory retention for offloaded information—termed the “Google effect” unless individuals maintain explicit intentions to remember (Grinschgl et al., 2021).

Despite the growing recognition of these phenomena, current assessment tools remain inadequate for measuring contemporary digital consumption patterns. Current digital addiction and problematic internet use scales face significant limitations when applied to contemporary content consumption patterns, particularly those involving short-form, algorithm-driven platforms. Most existing scales require further validation work and demonstrate inconsistent psychometric properties across different populations and contexts (Omrawo et al., 2023; Tiego et al., 2019), while traditional measures were primarily developed and validated for adult populations, leaving gaps in assessment tools for younger demographics.

Existing measures fail to capture the unique aspects of short-form content overconsumption because they were designed for longer-form digital engagement rather than the rapid, repetitive consumption cycles characteristic of platforms like TikTok (Griffiths, 2021; Kokshagina et al., 2023; Omrawo et al., 2023; Pastor, 2025). The advanced algorithmic systems that personalize content delivery create uniquely addictive patterns that differ substantially from traditional social media addiction, yet there remains an absence of valid and specific psychometric tools to assess these emerging forms of digital addiction (Kokshagina et al., 2023; Pastor, 2025).

To address these critical gaps in assessment capabilities, this study aims to develop and validate the Brain Rot Scale (BRS), a new tool designed to assess digital content overconsumption patterns among Generation Alpha and Generation Z. The BRS aims to capture the unique characteristics of contemporary short-form, algorithm-driven content consumption behaviors that existing digital addiction measures fail to adequately assess. Through rigorous psychometric evaluation, the study aims to establish the BRS as a specialized tool for measuring the cognitive, behavioral, and emotional consequences of excessive consumption of low-quality digital content. This research aims to provide researchers, clinicians, and educators with an empirically validated instrument to identify problematic digital consumption patterns among digital natives, addressing the critical gap in assessment tools for emerging forms of internet behavioral addiction.

### Literature Review

1. Transformation of Digital Media Consumption Patterns

The emergence of short-form video platforms has fundamentally transformed media consumption patterns among digital natives, creating a paradigmatic shift from traditional passive, scheduled viewing to active, algorithm-driven engagement characterized by continuous scrolling behaviors. This transformation represents more than a simple technological evolution; it constitutes a complete restructuring of how individuals interact with digital content and process information. Research shows that infinite scrolling mechanisms contribute to perceived loss of self-control and negative internal states, creating psychological experiences that users find difficult to regulate independently (Park & Jung, 2024). The behavioral patterns associated with these platforms are characterized by binge-scrolling behaviors that are driven by satisfaction and dependence on cognitive consumption values, establishing sustained engagement cycles that exceed users’ initial intentions and conscious awareness (Q. Zhang et al., 2024). These consumption patterns generate significant data inefficiencies, with substantial mobile data allocated to unwatched content (G. Zhang et al., 2023), while fostering addictive behaviors that raise concerns about user well-being and sustainable digital media engagement (Szalkowski et al., 2025; Q. Zhang et al., 2024). The technological architecture of contemporary platforms significantly impairs users’ capacity to retain behavioral intentions through constant context switching, creating cognitive disruption that undermines deliberate decision-making processes and goal-directed behavior (Chiossi et al., 2023). Research reveals that increased consumption of short-form content is linked to poor sustained attention, independent of consumption duration, suggesting that the rapid switching nature of content rather than time spent is the critical factor in attention degradation (Lin et al., 2024). The continuous engagement facilitated by these platforms is further reinforced by flow experiences and cognitive lock-in mechanisms that sustain user attention through serendipitous content discovery, creating a perpetual cycle of consumption that becomes increasingly difficult to voluntarily interrupt (Yang et al., 2023).

2. Neurobiological Mechanisms of Digital Platform Addiction

The addictive potential of digital platforms is grounded in well-established neurobiological mechanisms that demonstrate remarkable similarities to traditional substance addiction pathways. Digital platform addiction shares fundamental neurobiological mechanisms with traditional substance addiction, particularly through dopamine-mediated reward pathways and striatal circuits underlying craving and compulsive behavior (Antons et al., 2020; Weinstein & Lejoyeux, 2020). Both addiction types demonstrate heightened activation in reward regions like the ventral tegmental area and nucleus accumbens, alongside reduced activity in executive control areas, impairing decision-making and inhibitory control mechanisms essential for self-regulation (Poisson et al., 2021). The strategic implementation of intermittent reinforcement schedules through unpredictable digital rewards creates particularly potent activation of dopamine systems, establishing powerful conditioning mechanisms that drive continued engagement beyond conscious intention. Intermittent reinforcement from unpredictable digital rewards potently activates dopamine systems, while social prediction errors in the ventral tegmental area drive platform engagement through unexpected social feedback delivered through sophisticated algorithmic systems (Mestre-Bach & Potenza, 2023). This neurobiological process underlies the compelling nature of social media use, as dopamine-mediated reward learning drives users to maximize social reward acquisition by continuing to engage with the platform (Schultz, 2024). These neurobiological processes result in measurable neuroplastic changes that reflect the brain’s adaptation to digital engagement patterns. Neuroplastic changes, including reduced gray-matter volume and altered white-matter density, occur in both digital and substance addictions, though digital engagement uniquely affects sensory, motor, and cognitive processing regions (Dresp-Langley & Hutt, 2022). Research demonstrates that digital technology engagement produces structural changes, including increased gray matter volume in frontal regions associated with goal achievement and deduction (Hongo et al., 2023), while virtual reality-based cognitive training enhances neural activity by increasing alpha and beta EEG power (Gangemi et al., 2023). These neuroplastic adaptations reflect the brain’s dynamic reorganization of functional networks during human-machine interaction (T. Zhang et al., 2022), with gamified learning environments promoting cognitive enhancement and emotional regulation through network reorganization (Kumar et al., 2024).

3. Cognitive Offloading and Memory Consolidation Alterations

The phenomenon of cognitive offloading—defined as the use of external digital tools or actions to reduce mental effort in memory, navigation, and information retrieval tasks (Risko & Gilbert, 2016)—has fundamentally altered memory consolidation processes and cognitive development patterns, particularly among Generation Alpha and Generation Z who have integrated digital systems into their cognitive architecture from early developmental stages. This transformation is exemplified by the well-documented “Google effect,” in which individuals demonstrate significantly reduced retention of information they believe is reliably stored in external digital systems (Schooler & Storm, 2021). The decision-making process underlying cognitive offloading reflects sophisticated value-based metacognitive evaluation, in which users continuously balance cognitive effort expenditure with perceived memory utility and accessibility (Gilbert, 2024). The decision to engage in cognitive offloading is primarily driven by metacognitive evaluations of memory confidence rather than actual memory ability, with individuals demonstrating value-based decision-making processes when balancing cognitive effort against memory retention needs (Gilbert, 2024; X. Hu et al., 2019). While cognitive offloading can enhance immediate task performance, particularly under high cognitive load (Morrison & Richmond, 2020), and improve memory retention of non-offloaded information through “saving-enhanced memory” effects, excessive reliance on digital systems can contribute to a phenomenon termed “digital dementia.” This condition is characterized by attention deficits, memory impairment, and structural brain changes that occur during critical developmental periods when cognitive architecture is still forming (Ali et al., 2024). Empirical research indicates that 84.5% of students actively utilize smartphones as external memory systems, with significant correlations observed between this reliance and increased rates of depression, anxiety, and reduced emotional intelligence (Mohan & Ponnusamy, 2023; Musa & Ishak, 2021). This cognitive-efficiency trade-off underscores the complex metacognitive processes underlying digital dependency decisions among younger generations. It highlights the multifaceted nature of contemporary digital consumption, which extends beyond simple entertainment or communication.

4. Assessment Limitations and Measurement Gaps

Current assessment methodologies for digital consumption patterns demonstrate significant limitations when applied to contemporary short-form, algorithm-driven content engagement behaviors. Existing digital addiction and problematic internet use scales face significant limitations when applied to contemporary content consumption patterns, particularly those involving short-form, algorithm-driven platforms that operate through fundamentally different engagement mechanisms than traditional digital media (Griffiths, 2021; Kokshagina et al., 2023; Omrawo et al., 2023; Pastor, 2025). Most existing scales require extensive additional validation work and demonstrate inconsistent psychometric properties across different populations and contexts, while traditional measures were primarily developed and validated for adult populations, leaving significant gaps in assessment tools specifically designed for younger demographics who represent the primary consumers of contemporary digital content (Omrawo et al., 2023; Tiego et al., 2019). The rapid, repetitive consumption cycles characteristic of platforms like TikTok differ substantially from the longer-form digital engagement patterns for which current assessment tools were originally designed and validated. The advanced algorithmic systems that personalize content delivery create uniquely addictive patterns that differ substantially from traditional social media addiction mechanisms, yet there remains an absence of valid and specific psychometric tools to assess these emerging forms of digital behavioral addiction (Kokshagina et al., 2023; Pastor, 2025). Short video addiction represents an emerging form of internet behavioral addiction characterized by dependent and excessive use patterns that significantly affect decision-making processes, necessitating specialized measurement instruments that can accurately capture these contemporary digital consumption behaviors among Generation Alpha and Generation Z.

Neurobiological research, cognitive psychology, and cultural recognition of digital overconsumption phenomena have led to the development of specialized assessment tools. The “brain rot” phenomenon, characterized by cognitive decline and mental exhaustion from low-quality digital content, has gained attention due to its cultural resonance. This highlight concerns technology’s impact on cognitive health and meaningful communication, especially among younger generations who engage with algorithm-driven platforms. The lack of empirically validated assessment tools is a significant limitation in research and clinical practice. Based on the theoretical framework and the identified gap in measurement tools, the following hypotheses were formulated to guide the development and validation of the Brain Rot Scale (BRS):

− **H1:** *The Brain Rot Scale will demonstrate a multifactorial structure, reflecting the distinct yet related dimensions of digital content overconsumption.*
− **H2:** *The scale and its subscales demonstrate good internal consistency, with reliability indices (Cronbach’s alpha, McDonald’s omega, and Guttman’s lambda) exceeding the threshold of 0.70.*
− **H3:** *The factor structure identified through exploratory factor analysis will be confirmed, with the confirmatory factor analysis model demonstrating adequate fit to the data (CFI > 0.90, TLI > 0.90, RMSEA < 0.08).*

## 2. Materials and Methods

### 2.1. Participants

This study employed a two-phase design utilizing separate samples for exploratory factor analysis (EFA) and confirmatory factor analysis (CFA) to ensure robust psychometric validation of the Brain Rot Scale. The EFA sample comprised 403 Egyptian participants aged 8 to 24 years (*M* = 15.014, *SD* = 4.66), while the CFA sample comprised 897 Egyptian participants within the same age range (*M* = 17.035, *SD* = 3.954). Both samples were specifically selected to represent Generation Alpha (born after 2010) and Generation Z (born 1995–2010) populations, which constitute the primary target demographic for assessing digital content overconsumption behaviors. The demographic characteristics of both samples are presented in Table 1.

Table 1 shows that gender distribution is balanced, with males comprising 50.9% of the EFA sample and 53.0% of the CFA sample. Rural residents are predominant, accounting for 72.7% of the EFA sample and 72.0% of the CFA sample. Educational attainment varies between the samples, with primary school students being the largest group. Mobile phones are the predominant platform for digital content consumption, used by 66.0% of EFA participants and 63.9% of CFA participants. Multiple device usage is reported by 19.4% of EFA participants and 17.6% of CFA participants, while tablets, laptops, and computers show lower usage frequencies.

### 2.2. Instrument Development

The Brain Rot Scale (BRS) was developed to address the critical gap in psychometric instruments capable of assessing contemporary digital content overconsumption patterns among Generation Alpha and Generation Z. The initial instrument comprised 21 items specifically designed to capture the cognitive, behavioral, and emotional consequences associated with excessive consumption of low-quality digital content, particularly the short-form, algorithm-driven content characteristic of platforms. The scale prioritizes digital natives’ unique consumption behaviors.

The original scale was developed in Arabic for cultural and linguistic suitability, using a 5-point Likert format to measure digital content consumption behaviors. The instrument then underwent a professional translation into English by specialized translators with expertise in psychological assessment tools. The translation process employed forward and backward translation procedures to maintain semantic equivalence and conceptual integrity across languages. Content validity was established through expert review by specialists in educational psychology, clinical psychology, and mental health, who evaluated each item for relevance, clarity, and alignment with digital content overconsumption. Expert feedback was used to refine wording and ensure comprehensive coverage of the phenomenon.

### 2.3. Procedure

Data collection was conducted using digital survey platforms to effectively reach the target demographic of Generation Alpha and Generation Z participants. The recruitment strategy employed convenience sampling methods appropriate for accessing populations with high digital engagement. Participants were recruited through various digital channels, including social media platforms and educational institutions, to ensure diverse representation within the specified age range. The survey administration protocol was designed to minimize participant burden while ensuring comprehensive data collection for robust psychometric analysis.

The survey protocol included completion of the BRS items alongside demographic questionnaires capturing relevant participant characteristics. Demographic variables included age, gender, residence type (urban/rural), educational level, and primary device type used for digital content consumption. The inclusion of device type information was particularly important given the platform-specific nature of digital content overconsumption behaviors. Data collection was conducted in controlled conditions to minimize external influences on participant responses, with clear instructions provided for scale completion.

A convenience sampling strategy was employed to recruit participants through digital channels, including social media platforms (e.g., Facebook, WhatsApp groups) and with the cooperation of public and private schools. This method was deemed most appropriate for effectively reaching the target demographic of digitally native Generation Alpha and Generation Z individuals. Before participation, the study’s purpose and procedures were explained to all participants. For participants under 18, informed consent was obtained from a parent or legal guardian. Additionally, verbal or written assent was obtained from the minor participants themselves.

### 2.4. Inclusion and Exclusion Criteria

Participants were included if they were (a) within the age range of 8 to 24 years, (b) regular users of digital devices (defined as daily use for entertainment or social purposes), and (c) able to read and understand Arabic. Participants were excluded if they (a) had a self-reported diagnosis of a severe neurological or psychiatric disorder that could significantly impact cognitive function, or (b) were unable to complete the survey independently.”

### 2.5. Statistical Analysis

Data were analyzed using SPSS version 27 and AMOS version 26. Preliminary analyses included missing-data assessment (<2%), descriptive statistics, and normality testing. The Kaiser-Meyer-Olkin measure and Bartlett’s test of sphericity assessed factorability. Principal axis factoring with promax rotation was conducted on the EFA sample (*n* = 403), with factor retention guided by parallel analysis (1000 simulated datasets) comparing real eigenvalues to the 95th percentile of simulated eigenvalues. Items were retained based on primary loadings ≥0.50, cross-loadings <0.30, and communalities ≥0.40. Maximum likelihood confirmatory factor analysis with robust standard errors evaluated the factor structure in the independent CFA sample (*n* = 897). Model fit was assessed using multiple indices: CFI, TLI (≥0.90 acceptable), RMSEA (≤0.08 acceptable), SRMR (≤0.08 acceptable), and GFI (≥0.90 acceptable). Internal consistency was evaluated using McDonald’s omega, Cronbach’s alpha, and Guttman’s lambda. Convergent validity was assessed through composite reliability (CR ≥ 0.70) and average variance extracted (AVE ≥ 0.50). Statistical significance was set at *α* = 0.05.

## 3. Results

### 3.1. Preliminary Analyses and Data Screening

Prior to conducting factor analyses, data were screened for missing values, outliers, and distributional properties. Missing data was negligible (<2% across all items) and handled using listwise deletion. Descriptive statistics for the 21 initial items in the EFA sample (*n* = 403) revealed means ranging from 2.715 to 3.797 (*SD* = 0.940–1.328) on the 5-point Likert scale, with no evidence of floor or ceiling effects (Table 2). Skewness values ranged from −0.503 to 0.388, and kurtosis values ranged from −1.188 to 0.447, indicating acceptable univariate normality (skewness < 2.0, kurtosis < 7.0).

### 3.2. Factorability Assessment

The suitability of the correlation matrix for factor analysis was evaluated using multiple criteria. The Kaiser-Meyer-Olkin (KMO) measure of sampling adequacy was 0.883, classified as “meritorious” according to Kaiser’s (1974) guidelines and substantially exceeding the recommended minimum of 0.70 (Table 3). Individual item MSA values ranged from 0.785 to 0.938, with all items surpassing the 0.70 threshold, indicating that each item shared sufficient common variance with other items.

Bartlett’s test of sphericity yielded a highly significant result (χ^2^ = 2724.952, df = 210, *p* < 0.001), rejecting the null hypothesis that the correlation matrix was an identity matrix and confirming the presence of adequate inter-item correlations for factor extraction. Mardia’s multivariate normality test revealed significant departures from multivariate normality for both skewness (χ^2^ = 2822.314, df = 1771, *p* < 0.001) and kurtosis (z = 16.872, *p* < 0.001), justifying the use of polychoric correlations to account for the ordinal nature of Likert-scale responses.

### 3.3. Exploratory Factor Analysis

#### 3.3.1. Extraction Method and Factor Retention

Principal Axis Factoring (PAF) with promax rotation (κ = 4) was conducted on the polychoric correlation matrix of the 21 initial items. PAF was selected over principal components analysis because it focuses on extracting common variance attributable to latent factors rather than maximizing total variance explanation, making it more appropriate for scale development (Costello & Osborne, 2005). Promax rotation was chosen based on the theoretical expectation that dimensions of digital overconsumption would be intercorrelated rather than orthogonal, consistent with prior research on related constructs (Montag & Walla, 2016).

Factor retention was determined through triangulation of multiple criteria: the Kaiser-Guttman rule (eigenvalues > 1.0), parallel analysis based on 1000 randomly generated datasets, and visual inspection of the scree plot. The initial extraction yielded six factors with eigenvalues exceeding 1.0 (λ_1_ = 6.145, λ_2_ = 1.919, λ_3_ = 1.817, λ_4_ = 1.072, λ_5_ = 1.012, λ_6_ = 0.945). However, parallel analysis—considered a more robust criterion (Hayton et al., 2004; Horn, 1965)—indicated that only three factors had eigenvalues from the real data exceeding the 95th percentile of eigenvalues from simulated random data (Table 4).

**Figure 1:**
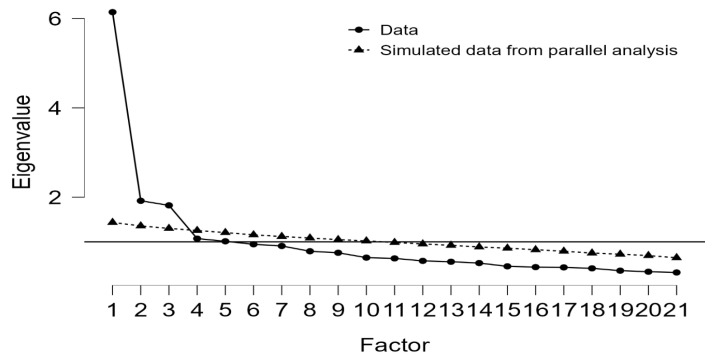
Scree Plot with Parallel Analysis Comparison for Factor Retention. Note. The solid line represents eigenvalues from the actual data; the dashed line represents the 95th percentile of eigenvalues from 1000 randomly generated datasets with equivalent dimensions. Factors with actual eigenvalues exceeding the simulated threshold (Factors 1–3) were retained. The pronounced elbow after Factor 3 further supports the three-factor solution.

Visual inspection of the scree plot (Figure 1) corroborated the parallel analysis findings, revealing a pronounced elbow after the third factor, with subsequent factors contributing marginally to total variance. The convergence of these retention criteria provided strong empirical support for a three-factor solution.

#### 3.3.2. Item Retention and Factor Structure

The three-factor PAF solution was extracted with promax rotation. Items were evaluated for retention based on multiple criteria: (a) primary factor loadings ≥0.50 on their designated factor, (b) cross-loadings <0.30 on non-target factors, (c) communalities ≥0.40, and (d) conceptual coherence with the intended factor (Comrey & Lee, 1992; Tabachnick & Fidell, 2013). Seven items (Items 4, 5, 6, 13, 14, 20, and 21) failed to meet these criteria and were removed iteratively. Items 4, 5, and 6 exhibited low communalities (<0.20) and weak primary loadings (<0.40). Items 13 and 14 demonstrated substantial cross-loadings (>0.35) on multiple factors. Items 20 and 21 showed primary loadings below 0.50 (0.452 and indeterminate, respectively) and problematic cross-loadings.

The final three-factor solution with 14 items accounted for 35.114% of the common variance after rotation, with individual factors explaining 23.679% (Factor 1), 5.854% (Factor 2), and 5.581% (Factor 3) of variance (Table 5). Although the total variance explained may appear modest by conventional standards (<50%), it is consistent with complex psychological constructs in social sciences (Henson & Roberts, 2006) and substantially improved from the initial 39.673% total variance (which included specific and error variance under PCA).

#### 3.3.3. Pattern Matrix and Factor Interpretation

The pattern matrix presents standardized regression coefficients representing the unique contribution of each factor to item variance, controlling for other factors (Table 6). Factor 1, labeled Attention Dysregulation (AD), comprised six items (loadings ranging from 0.581 to 0.735) that capture difficulties with sustained attention, task completion, focus maintenance, and cognitive control in digital environments. This dimension reflects impaired attentional capacity, manifested as frequent task-switching, difficulty concentrating without digital stimulation, and mind-wandering during cognitively demanding activities.

Factor 2, labeled Digital Compulsivity (DC), included five items (loadings from 0.539 to 0.750) that measure compulsive device-checking behaviors, unsuccessful attempts at screen time regulation, immediate responsiveness to digital notifications, and anxiety associated with device separation or connectivity loss. This dimension captures the behavioral and affective components of problematic digital engagement characterized by loss of volitional control.

Factor 3, labeled Cognitive Dependency (CD), contained three items (loadings from 0.610 to 0.719) that assess reliance on digital devices for basic cognitive functions traditionally performed by biological memory and spatial navigation systems. This dimension reflects the externalization of cognitive processes and diminished confidence in internal cognitive resources.

The structure matrix revealed some notable secondary loadings, particularly for Item 8 (AD) and Item 18 (DC), which showed cross-loadings with other factors. However, all primary loadings substantially exceeded secondary loadings (Δλ > 0.20), supporting the simple structure criterion (Thurstone, 1947) and the conceptual distinctiveness of the three factors.

Communalities ranged from 0.305 (Item 7) to 0.539 (Item 19), indicating that 30.5% to 53.9% of item variance was explained by the common factors. While some communalities were below the idealized threshold of 0.50, they exceeded the minimum acceptable level of 0.30 (Costello & Osborne, 2005), and strong primary loadings and conceptual relevance justified their retention.

#### 3.3.4. Factor Intercorrelations

The factor correlation matrix revealed moderate-to-strong positive correlations among the three factors, ranging from 0.404 to 0.548 (Table 7). These intercorrelations justified the use of oblique rotation and indicated that while the dimensions are empirically distinguishable, they represent related aspects of a broader construct. The strongest correlation was between AD and DC (r = 0.548), suggesting that attentional impairments and compulsive behaviors may be reciprocally reinforcing. The correlations between CD and the other two factors were somewhat lower (r = 0.404–0.406), indicating greater distinctiveness of this dimension.

### 3.4. Confirmatory Factor Analysis

#### 3.4.1. Sample Characteristics for CFA

The three-factor structure identified through EFA was tested using confirmatory factor analysis on an independent validation sample (*n* = 897). Descriptive statistics for the 14 items in the CFA sample (Table 8) revealed means ranging from 2.841 to 3.530 (*SD* = 1.150–1.360), consistent with the EFA sample and indicating no evidence of systematic differences between samples. Skewness values ranged from −0.503 to 0.063, and kurtosis values from −1.098 to −0.621, all within acceptable limits for maximum likelihood estimation with robust standard errors.

#### 3.4.2. Model Specification and Estimation

Two theoretically plausible models were evaluated: (1) a first-order three-factor correlated model in which AD, DC, and CD are allowed to covary freely, and (2) a second-order hierarchical model in which a general “Brain Rot” factor loads onto the three first-order dimensions. Maximum likelihood estimation with robust standard errors (MLR) was employed to accommodate potential violations of multivariate normality. All items were specified as continuous indicators of their respective latent factors, with factor loadings freely estimated except for reference indicators (fixed to 1.0 for scale identification). Error variances were constrained to be non-negative, and no correlated errors were specified a priori.

#### 3.4.3. First-Order Three-Factor Model

The first-order correlated three-factor model demonstrated excellent fit to the data across multiple absolute, incremental, and parsimonious fit indices (Table 9). The chi-square test was statistically significant (χ^2^ = 137.549, df = 74, *p* < 0.001), which is expected given the large sample size (*n* = 897) and the sensitivity of this omnibus test to trivial misspecifications (Barrett, 2007; Bentler & Bonett, 1980). More informative is the normed chi-square ratio (CMIN/DF = 1.859), which fell well below the conservative threshold of 3.0 and approached the stringent criterion of 2.0, indicating excellent model parsimony relative to fit (Kline, 2016).

The Comparative Fit Index (CFI = 0.988) and Tucker-Lewis Index (TLI = 0.985) substantially exceeded the conventional threshold of 0.90 and approached the stringent criterion of 0.95, indicating that the specified model explained 98.8% and 98.5%, respectively, of the covariation in the data relative to a null independence model (L. Hu & Bentler, 1999). The Incremental Fit Index (IFI = 0.988), Normed Fit Index (NFI = 0.973), and Relative Fit Index (RFI = 0.967) similarly demonstrated excellent incremental fit, with the RFI—which had been noted as a potential concern in preliminary analyses due to its penalty for model complexity—comfortably exceeding the 0.90 criterion.

The Root Mean Square Error of Approximation (RMSEA = 0.031, 90% CI [0.023, 0.039]) fell well below the stringent threshold of 0.05, with the upper bound of the confidence interval remaining below 0.04, indicating “close fit” according to Browne and Cudeck’s (1993) criteria. The Standardized Root Mean Square Residual (SRMR = 0.040) was also below the recommended threshold of 0.08, indicating minimal average discrepancy between observed and model-implied correlations (L. Hu & Bentler, 1999).

Absolute fit indices further supported model adequacy. The Goodness-of-Fit Index (GFI = 0.979) and Adjusted Goodness-of-Fit Index (AGFI = 0.970) both exceeded 0.90, indicating that the model accounted for 97.9% and 97.0%, respectively, of the observed variance-covariance matrix, with the AGFI adjusting for model complexity. Parsimonious fit indices (PNFI = 0.792, PCFI = 0.803) exceeded the minimum threshold of 0.50, suggesting that the model achieved good fit without excessive parameterization (Mulaik et al., 1989).

#### 3.4.4. Standardized Parameter Estimates

**Figure 2:**
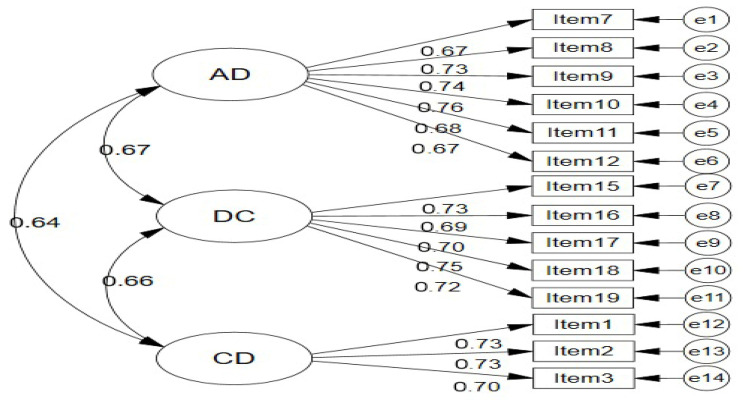
Standardized First-Order Three-Factor CFA Model of BRS-14.

All standardized factor loadings were statistically significant (*p* < 0.001) and ranged from 0.667 to 0.758, substantially exceeding the minimum threshold of 0.40 and approaching or exceeding the ideal benchmark of 0.70. These loadings indicate that each item is strong indicator of its intended latent construct, with 44.5% to 57.5% of item variance (R^2^) explained by the underlying factors (Figure 2).

Factor covariances (converted to correlations) ranged from 0.636 to 0.671, indicating substantial positive relationships among the three dimensions. The strongest correlation was between AD and DC (r = 0.671), consistent with EFA findings, suggesting that attentional impairments and compulsive behaviors are closely intertwined. CD showed slightly lower correlations with AD (r = 0.636) and DC (r = 0.663), supporting its relative distinctiveness while confirming its membership in the broader nomological network.

#### 3.4.5. Second-Order Hierarchical Model

**Figure 3:**
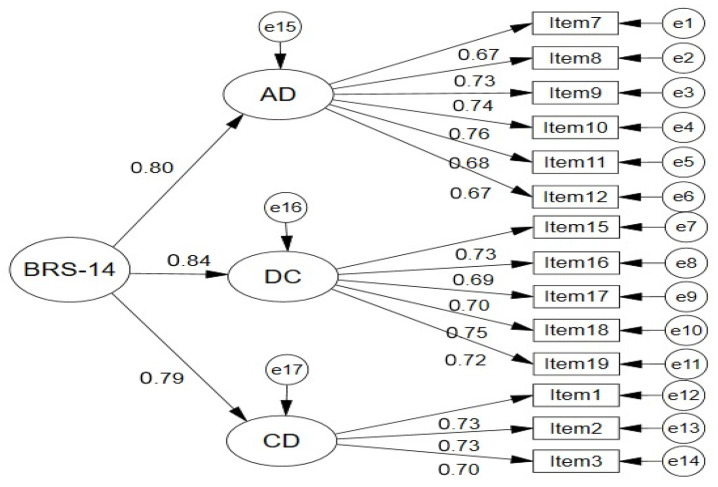
Standardized Second-Order Hierarchical CFA Model of BRS-14 (*n* = 897).

A second-order model was specified to test the hypothesis that the three first-order dimensions (AD, DC, CD) are manifestations of a single superordinate construct labeled “Brain Rot.” In this model, a general second-order factor loads onto the three first-order factors, which in turn load onto their respective observed indicators (Figure 3). Mathematically, this model is equivalent to the first-order correlated model when no additional constraints are imposed (i.e., both have 74 degrees of freedom), resulting in identical fit indices (χ^2^ = 137.549, df = 74, all fit indices identical to Table 8).

However, the second-order parameterization offers theoretical advantages by explicitly modeling the hierarchical structure and partitioning variance into general (second-order) and specific (first-order) components. Second-order standardized loadings indicated that the general Brain Rot factor explained substantial variance in all three dimensions: 64.3% in AD (λ = 0.802), 69.9% in DC (λ = 0.836), and 62.9% in CD (λ = 0.793). These loadings suggest that while the dimensions retain specific variance (35.7%, 30.1%, and 37.1%, respectively), they share considerable common variance attributable to a unitary higher-order construct.

### 3.5. Reliability Analysis

#### Internal Consistency Coefficients

Internal consistency reliability was evaluated using three complementary coefficients: McDonald’s omega (*ω*), Cronbach’s alpha (*α*), and Guttman’s lambda-2 (λ_2_). Omega was prioritized as it provides a model-based estimate of reliability that accounts for differential item contributions (factor loadings) and does not assume tau-equivalence (equal loadings), making it superior to alpha for congeneric measures (Dunn et al., 2013; McDonald, 1999). All coefficients were computed with 95% confidence intervals via bootstrapping (10,000 resamples).

The overall 14-item BRS demonstrated excellent reliability across all indices (Table 10): *ω* = 0.900 (95% CI [0.890, 0.909]), *α* = 0.899 (95% CI [0.886, 0.912]), and λ_2_ = 0.901 (95% CI [0.889, 0.914]). All estimates substantially exceeded the 0.70 threshold for adequate reliability and approached the 0.90 criterion for excellent reliability (Nunnally & Bernstein, 1994). The narrow confidence intervals indicate high precision of these estimates.

Subscale reliability ranged from good to excellent. The AD subscale (6 items) demonstrated excellent consistency (*ω* = 0.857, *α* = 0.857, λ_2_ = 0.857), as did the DC subscale (5 items: *ω* = 0.844, *α* = 0.844, λ_2_ = 0.844). The CD subscale (3 items) showed good reliability (*ω* = 0.759, *α* = 0.759, λ_2_ = 0.759), which is commendable given the brevity of this dimension and approaches the threshold for “good” reliability.

### 3.6. Convergent Validity

Convergent validity—the extent to which indicators of the same construct converge or share high proportions of common variance—was assessed through three indices: Composite Reliability (CR), Average Variance Extracted (AVE), and Maximum Reliability (MaxR(H)). Fornell and Larcker (1981) recommend CR > 0.70 and AVE > 0.50 as evidence of adequate convergent validity.

All three subscales met or exceeded these criteria (Table 11). CR values ranged from 0.764 (CD) to 0.857 (AD), all surpassing the 0.70 threshold. AVE values ranged from 0.501 (AD) to 0.521 (DC), exceeding the 0.50 criterion and indicating that on average, more than 50% of item variance is explained by the underlying construct (with the remainder attributable to measurement error and item-specific variance). MaxR(H) an asymptotic estimate of the maximum possible reliability given observed item intercorrelations—ranged from 0.764 to 0.860, suggesting that reliability estimates are near their theoretical maximum and that additional items would yield minimal reliability gains.

The second-order model yielded even stronger convergent validity indices (CR = 0.852, AVE = 0.657), indicating that 65.7% of variance in the three first-order factors is explained by the general Brain Rot construct. This substantial shared variance supports the theoretical coherence of the overarching construct while acknowledging dimension-specific variance (34.3%).

The BRS-14 demonstrated robust psychometric properties (Appendix A). The three-factor structure was supported through PAF with parallel analysis and confirmed via CFA with excellent fit. The scale showed excellent reliability (*ω* = 0.900), and adequate convergent validity (CR > 0.70, AVE > 0.50). Factor loadings ranged from 0.667 to 0.758, all statistically significant. The moderate factor intercorrelations (0.636–0.671) and strong second-order loadings (0.793–0.836) support a hierarchical conceptualization with three distinguishable yet related dimensions of digital overconsumption.

## 4. Discussion

The development and validation of the Brain Rot Scale (BRS-14) represents a significant advancement in the assessment of digital content overconsumption among Generation Alpha and Generation Z. The rigorous psychometric evaluation conducted across two independent samples demonstrates that the BRS-14 is a reliable and valid instrument capable of capturing the unique characteristics of contemporary digital media consumption behaviors that existing measures have failed to adequately assess (Omrawo et al., 2023; Griffiths, 2021; Kokshagina et al., 2023).

The three-factor structure that emerged from our analyses—AD, DC, and CD—provides a comprehensive framework for understanding the multidimensional nature of digital content overconsumption. This structure aligns with theoretical conceptualizations of problematic digital media use while addressing the specific cognitive, behavioral, and emotional consequences associated with excessive consumption of short-form, algorithm-driven content.

The AD factor captures the fundamental cognitive disruptions that characterize brain rot phenomena. The items loading on this factor reflect difficulties with sustained attention, focus maintenance, and cognitive control that are increasingly prevalent among digital natives. These findings are consistent with neuroscience research demonstrating that excessive digital stimulation is associated with impaired learning and memory acquisition while potentially altering brain structure and function, particularly in regions responsible for executive function (Neophytou et al., 2019; Nivins et al., 2024; Small et al., 2022). The emergence of AD as a distinct factor is consistent with growing concerns about how constant exposure to rapid-fire digital content may be eroding cognitive capacities essential for deep learning and meaningful engagement with complex information (Yousef et al., 2025; Sage, 2025).

DC, as the second factor, encompasses behavioral manifestations of problematic digital engagement, including compulsive checking, failed attempts at reducing screen time, and immediate responsiveness to digital notifications. This dimension may reflect the neurobiological processes underlying social media addiction, in which platforms strategically employ intermittent reinforcement schedules by algorithmically delivering unpredictable rewards (Lindström et al., 2021). The factor structure is consistent with concerns that these variable reinforcement patterns may engage midbrain-limbic dopamine systems, potentially creating patterns similar to those observed in substance addiction (De et al., 2025; Solié et al., 2022; J. Wang & Wang, 2025). The identification of phantom vibration syndrome—a phenomenon where individuals perceive vibrations from a phone that is not actually vibrating—as a component of DC particularly underscores the extent to which digital dependency has become embodied in users’ sensory experiences.

The CD factor addresses perhaps the most concerning aspect of brain rot: the replacement of traditional cognitive processes with digital alternatives. Items loading on this factor reveal how users increasingly rely on external digital sources for basic cognitive functions, from memory storage to navigation and information retrieval. This finding is consistent with research on cognitive offloading, where the use of external tools reduces mental effort but may potentially diminish memory retention for offloaded information—termed the “Google effect” (Risko & Gilbert, 2016; Grinschgl et al., 2021). The emergence of CD as a distinct factor suggests that digital content overconsumption may be fundamentally altering how individuals process, store, and retrieve information, with potential implications for cognitive autonomy and intellectual development (Gilbert, 2024; Morrison & Richmond, 2020).

The strong inter-factor correlations observed in our study indicate that these three dimensions, while distinct, are interconnected aspects of a broader phenomenon. This interconnectedness suggests that digital content overconsumption operates as a systemic issue affecting multiple cognitive and behavioral domains simultaneously. The moderate to strong correlations between factors supports the conceptualization of brain rot as a multidimensional construct while maintaining the utility of examining its component dimensions separately.

The excellent psychometric properties of the BRS-14, including high internal consistency, reliability and strong model fit indices, establish confidence in the instrument’s measurement precision. The scale’s convergent validity, demonstrated through appropriate composite reliability and average variance extracted values, confirms that the factors adequately capture their intended constructs. These psychometric strengths are particularly important given the novelty of the brain rot construct and the need for precise measurement tools in this emerging area of research.

The cultural and linguistic development of the BRS-14 in Arabic, with subsequent professional translation to English, addresses important considerations of cultural validity in psychological assessment. Successful validation across these linguistic contexts suggests that brain rot phenomena may reflect universal concerns about digital technology’s impact on cognitive functioning. This cross-linguistic validity enhances the potential for international research collaboration and comparative studies across different cultural contexts.

The demographic characteristics of our samples, particularly the predominance of rural participants and the diversity of educational levels, strengthen the generalizability of our findings beyond urban, highly educated populations. The prevalence of mobile phone usage among participants aligns with global trends in digital media consumption. It ensures that our instrument captures behaviors relevant to the primary platforms where brain rot phenomena occur.

Methodologically, the study has several limitations that further contextualize the findings. The use of a non-probability convenience sampling approach, although effective for initial scale development, may have introduced selection bias and limited the generalizability of the results, particularly due to an overrepresentation of rural participants. Furthermore, while the psychometric validation revealed a robust three-factor structure, it was constrained by an explained variance of 39.7% and the lack of evidence for concurrent and discriminant validity. The relationship between the scale and established digital addiction metrics remains unverified, and the absence of clinically validated cut-off points confines the scale’s application primarily to research contexts for assessing underlying trait levels rather than clinical diagnosis.

The clinical and educational implications of BRS-14 are substantial. For clinicians, the scale provides a standardized tool for identifying problematic digital consumption patterns that may require intervention. The multidimensional structure allows for targeted assessment of specific problem areas, informing personalized treatment approaches. Educators can utilize the BRS-14 to identify students at risk for digital-related cognitive difficulties and implement appropriate support strategies.

The development of intervention strategies targeting the specific dimensions identified in the BRS-14 represents another crucial research direction. Attention training programs, digital wellness interventions, and cognitive rehabilitation approaches could be evaluated using the BRS-14 as an outcome measure. The scale’s sensitivity to change over time should be established through longitudinal intervention studies.

## 5. Conclusions

In conclusion, the development and initial validation of the Brain Rot Scale (BRS-14) provide a preliminary yet crucial step toward assessing contemporary digital content overconsumption among digital natives. This study establishes the BRS-14 as a psychometrically sound instrument in its early stages, demonstrating a clear three-factor structure, good internal consistency, and adequate model fit within the specific context of its development. These findings suggest that the construct of “brain rot,” characterized by AD, DC, and CD, is a measurable phenomenon that warrants further scholarly investigation. The BRS-14 thus offers researchers a foundational tool for exploring the cognitive and behavioral consequences of short-form, algorithm-driven media. However, its current status as an initially validated scale must be emphasized. Future research is imperative to cross-validate the factor structure in more diverse and representative populations, establish clinical cut-off scores, and rigorously test its concurrent and predictive validity against behavioral measures and existing digital addiction scales.

## Figures and Tables

**Table 1:** Demographic Characteristics of the participants.

Variable	Category	EFA Sample		CFA Sample	
		N	%	N	%
Gender	Male	205	50.9	475	53.0
	Female	198	49.1	422	47.0
Residence	Urban	110	27.3	251	28.0
	Rural	293	72.7	646	72.0
Educational Level	University	145	36.0	336	37.5
	Secondary	36	8.6	216	24.1
	Preparatory	22	5.5	228	25.4
	Primary	200	49.6	117	13.0
Device Type Used	Phone	266	66.0	573	63.9
	Tablet	29	7.2	49	5.5
	Laptop	28	6.9	63	7.0
	Computer	2	5	54	6.0
	Two devices or more	78	19.4	158	17.6

**Table 2:** Descriptive Statistics for Initial 21-Item Pool.

Item	*M*	*SD*	Skewness	Kurtosis
Item 1	3.238	1.080	−0.289	−0.644
Item 2	3.141	1.045	−0.208	−0.680
Item 3	3.251	1.283	−0.274	−1.116
Item 4	3.412	1.092	−0.407	−0.664
Item 5	3.797	0.940	−0.503	−0.488
Item 6	2.715	1.278	0.199	−1.098
Item 7	3.104	1.176	−0.165	−1.014
Item 8	2.901	1.281	0.032	−1.104
Item 9	3.231	1.182	−0.267	−0.919
Item 10	3.102	1.132	−0.181	−0.824
Item 11	2.938	1.171	0.044	−0.912
Item 12	3.077	1.118	−0.123	−0.801
Item 13	3.288	1.028	−0.325	−0.541
Item 14	2.844	1.275	0.082	−1.052
Item 15	3.640	1.229	−0.527	−0.811
Item 16	3.261	1.258	−0.312	−1.024
Item 17	3.132	1.242	−0.188	−0.986
Item 18	3.417	1.254	−0.436	−0.855
Item 19	3.263	1.234	−0.282	−1.002
Item 20	2.737	1.260	0.169	−1.034
Item 21	3.020	1.293	−0.062	−1.188

**Table 3:** Preliminary Tests for Factor Analytic Suitability.

Test	Statistic	df	Value	*p*
Kaiser-Meyer-Olkin (KMO)	Overall MSA	-	0.883	-
	Range of individual MSA	-	0.785–0.938	-
Bartlett’s Test of Sphericity	χ^2^	210	2724.952	<0.001
Mardia’s Test	Skewness (χ^2^)	1771	2822.314	<0.001
	Kurtosis (z)	-	16.872	<0.001

Note. MSA = Measure of Sampling Adequacy. KMO interpretation: 0.90+ = marvelous, 0.80–0.89 = meritorious, 0.70–0.79 = middling, 0.60–0.69 = mediocre, 0.50–0.59 = miserable, <0.50 = unacceptable (Kaiser, 1974).

**Table 4:** Parallel Analysis Results for Factor Retention.

Component	Real Data Eigenvalue	Simulated Mean Eigenvalue	95th Percentile	Decision
1	6.145	1.364	1.430	Retain *
2	1.919	1.320	1.354	Retain *
3	1.817	1.283	1.301	Retain *
4	1.072	1.251	1.251	Reject
5	1.012	1.221	1.204	Reject
6	0.945	1.192	1.158	Reject
7	0.907	1.165	1.121	Reject
8	0.789	1.139	1.090	Reject
9	0.755	1.114	1.051	Reject
10	0.646	1.090	1.019	Reject

Note. Parallel analysis based on 1000 random datasets with same dimensions (403 cases × 21 variables). * Factor retained when real data eigenvalue exceeds 95th percentile of simulated eigenvalues. Extraction method: Principal Components (for parallel analysis comparison).

**Table 5:** Total Variance Explained by Three-Factor PAF Solution.

Factor	Initial Eigenvalues			Extraction Sums of Squared Loadings			Rotation Sums of Squared Loadings
	Total	% Variance	Cumulative %	Total	% Variance	Cumulative %	Total
1	6.145	29.263	29.263	4.973	23.679	23.679	4.075
2	1.919	9.138	38.401	1.229	5.854	29.533	3.925
3	1.817	8.651	47.052	1.172	5.581	35.114	2.699

**Table 6:** Pattern Matrix and Structure Matrix from Promax-Rotated Three-Factor Solution.

Item	Pattern Coefficients			Structure Coefficients			h^2^	u^2^
	F1 (AD)	F2 (DC)	F3 (CD)	F1 (AD)	F2 (DC)	F3 (CD)		
Item 10: I frequently switch between multiple digital activities or applications without completing tasks.	0.735	-	-	0.720	0.418	-	0.531	0.469
Item 9: When reading longer texts, I find myself skimming rather than reading thoroughly and often lose track of the content.	0.676	-	-	0.668	-	-	0.446	0.554
Item 12: I find it challenging to follow complex verbal instructions without visual aid or having to ask for repetition.	0.656	-	-	0.613	-	-	0.380	0.620
Item 11: I feel restless or irritable when engaged in activities that require sustained attention without digital stimulation.	0.621	-	-	0.593	-	-	0.354	0.646
Item 7: I need background digital stimulation (music, videos, social media) to complete routine tasks that require minimal mental effort.	0.581	-	-	0.550	-	-	0.305	0.695
Item 8: I have difficulty maintaining focus on a single task for more than 15–20 min without checking my phone or other devices.	0.553	-	-	0.647	0.447	-	0.437	0.563
Item 19: I check notifications immediately when they appear, even during important conversations or activities.	-	0.750	-	-	0.733	-	0.539	0.461
Item 16: I feel anxious or uncomfortable when my battery is low or when I have no internet connection.	-	0.705	-	-	0.658	-	0.445	0.555
Item 18: I frequently find myself using digital devices for longer periods than I initially intended.	-	0.650	-	0.433	0.667	-	0.467	0.533
Item 15: I check my device immediately upon waking and right before sleeping, even when there is no specific reason to do so.	-	0.646	-	-	0.658	-	0.436	0.564
Item 17: I have tried to reduce my screen time but consistently fail to meet my goals.	-	0.539	-	0.428	0.625	-	0.406	0.594
Item 2: When asked a question I do not immediately know the answer to, my first instinct is to search online rather than try to remember.	-	-	0.719	-	-	0.686	0.475	0.525
Item 1: I struggle to recall basic information (phone numbers, addresses, dates) without checking my digital devices.	-	-	0.617	-	-	0.601	0.365	0.635
Item 3: I experience anxiety when separated from my devices because they contain information I need to function.	-	-	0.610	-	-	0.653	0.449	0.551

Note. h^2^ = communality (common variance); u^2^ = uniqueness (specific + error variance). Extraction method: Principal Axis Factoring. Rotation: Promax with Kaiser normalization (κ = 4).

**Table 7:** Factor Correlation Matrix from Promax Rotation.

Factor	1	2	3
1. AD	1		
2. DC	0.548 ***	1	
3. CD	0.406 ***	0.404 ***	1

Note. *** *p* < 0.001.

**Table 8:** Descriptive Statistics for BRS-14 Items in CFA Sample (*n* = 897).

Item	*M*	*SD*	Skewness	Kurtosis
**F1 (AD)**				
Item 7	2.915	1.275	−0.126	−1.038
Item 8	2.855	1.318	0.046	−1.093
Item 9	3.125	1.268	−0.255	−0.895
Item 10	2.990	1.228	−0.155	−0.865
Item 11	2.841	1.242	0.063	−0.901
Item 12	2.912	1.206	−0.075	−0.841
**F2 (DC)**				
Item 15	3.530	1.355	−0.503	−0.926
Item 16	3.237	1.360	−0.321	−1.079
Item 17	3.061	1.289	−0.136	−1.001
Item 18	3.332	1.287	−0.445	−0.796
Item 19	3.204	1.330	−0.235	−1.039
**F3 (CD)**				
Item 1	3.162	1.187	−0.295	−0.621
Item 2	3.105	1.154	−0.214	−0.665
Item 3	3.122	1.355	−0.227	−1.098

**Table 9:** Goodness-of-Fit Indices for First-Order and Second-Order CFA Models.

Fit Index	First-Order Model	Second-Order Model	Acceptable Threshold
**Absolute Fit**			
χ^2^ (*p*-value)	137.549 (<0.001)	137.549 (<0.001)	*p* > 0.05
df	74	74	-
CMIN/DF	1.859	1.859	<3.0 (<2.0 ideal)
GFI	0.979	0.979	>0.90
AGFI	0.970	0.970	>0.90
SRMR	0.040	0.040	<0.08
RMSEA	0.031	0.031	<0.08 (<0.05 ideal)
RMSEA 90% CI	[0.023, 0.039]	[0.023, 0.039]	Upper < 0.08
**Incremental Fit**			
CFI	0.988	0.988	>0.90 (>0.95 ideal)
TLI (NNFI)	0.985	0.985	>0.90 (>0.95 ideal)
IFI	0.988	0.988	>0.90
NFI	0.973	0.973	>0.90
RFI	0.967	0.967	>0.90

Note. GFI = Goodness-of-Fit Index; AGFI = Adjusted Goodness-of-Fit Index; SRMR = Standardized Root Mean Square Residual; RMSEA = Root Mean Square Error of Approximation; CI = Confidence Interval; CFI = Comparative Fit Index; TLI = Tucker-Lewis Index; NNFI = Non-Normed Fit Index; IFI = Incremental Fit Index; NFI = Normed Fit Index; RFI = Relative Fit Index.

**Table 10:** Reliability Coefficients for BRS-14 Total Scale and Subscales (*n* = 897).

Scale	Items N	*ω*	*α*	*λ*_2_
AD	6	0.857	0.857	0.857
DC	5	0.844	0.844	0.844
CD	3	0.759	0.759	0.759
Overall BRS-14	14	0.900	0.899	0.901

**Table 11:** Convergent Validity Indices for BRS-14 Subscales (*n* = 897).

Subscale	k	CR	AVE	MaxR(H)
AD	6	0.857	0.501	0.860
DC	5	0.845	0.521	0.846
CD	3	0.764	0.518	0.764
Overall (Second-Order)	14	0.852	0.657	0.853

## Data Availability

The datasets generated and analyzed during the current study are available from the corresponding authors upon reasonable request.

## Abbreviations

The following abbreviations are used in this manuscript:

BRS	Brain Rot Scale
CFA	confirmatory factor analysis
EFA	exploratory factor analysis

## Appendix A

**The Brain Rot Scale (BRS-14)**

**Instructions**

This questionnaire assesses your experiences with digital devices (smartphones, tablets, computers) and digital content (social media, videos, games, websites). Please read each statement carefully and indicate how much you agree or disagree with it based on your typical experiences over the past month.

There are no right or wrong answers. Please respond honestly based on your actual behavior and feelings.

**Response Options**

For each statement below, select the number that best describes your experience:

1	2	3	4	5
**Strongly Disagree**	**Disagree**	**Neither Agree nor Disagree**	**Agree**	**Strongly Agree**

**Scale Items**

**Factor 1: Attention Dysregulation (AD)**

I need background digital stimulation (music, videos, social media) to complete routine tasks that require minimal mental effort.

I have difficulty maintaining focus on a single task for more than 15–20 min without checking my phone or other devices.

When reading longer texts, I find myself skimming rather than reading thoroughly and often lose track of the content.

I frequently switch between multiple digital activities or applications without completing tasks.

I feel restless or irritable when engaged in activities that require sustained attention without digital stimulation.

I find it challenging to follow complex verbal instructions without visual aid or having to ask for repetition.

**Factor 2: Digital Compulsivity (DC)**

I check my device immediately upon waking and right before sleeping, even when there is no specific reason to do so.

I feel anxious or uncomfortable when my battery is low or when I have no internet connection.

I have tried to reduce my screen time but consistently fail to meet my goals.

I frequently find myself using digital devices for longer periods than I initially intended.

I check notifications immediately when they appear, even during important conversations or activities.

**Factor 3: Cognitive Dependency (CD)**

I struggle to recall basic information (phone numbers, addresses, dates) without checking my digital devices.

When asked a question I do not immediately know the answer to, my first instinct is to search online rather than try to remember.

I experience anxiety when separated from my devices because they contain information I need to function.
